# Relation between proteinuria and acute kidney injury in patients with severe burns

**DOI:** 10.1186/cc11649

**Published:** 2012-09-29

**Authors:** Jiong Yu Hu, Xin Chun Meng, Jian Han, Fei Xiang, Ya Dong Fang, Jun Wu, Yi Zhi Peng, Ya Zhou Wu, Yue Sheng Huang, Qi Zhi Luo

**Affiliations:** 1 Institute of Burn Research, Southwest Hospital, State Key Laboratory of Trauma, Burns and Combined Injury, Chongqing Key Lab for Disease Proteomics, Third Military Medical University, Chongqing 400038, PR China; 2 Burn Department, People Hospital of Changshou District, Chongqing 401220, PR China; 3 Department of Gynecology and Obstetrics, Daping Hospital, Third Military Medical University, Chongqing 400042, PR China; 4 Department of Statistics, Third Military Medical University, Chongqing 40038, PR China

**Keywords:** Severe burn, Proteinuria, Acute kidney injury, Risk factors, Mortality

## Abstract

**Introduction:**

Proteinuria in burn patients is common, and may be associated with acute kidney injury (AKI) and adverse outcomes. We evaluated the incidences, outcomes, characteristics and determinants of proteinuria and its influence on AKI and outcomes in burn patients.

**Methods:**

This retrospective study was carried out in a hospital's burn department. The study population consisted of patients with burn injuries admitted during a five-year period. Positive urine dipstick readings were defined as mild (± or 1+) or heavy (≥ 2+) proteinuria, and AKI was diagnosed and staged according to the Risk, Injury, Failure, Loss, End Stage (RIFLE) classification system. Patient characteristics, management and outcomes were evaluated for associations with proteinuria using nonparametric tests, chi-square (χ^2^) tests and binary logistic regression.

**Results:**

Of the patients admitted to the burn unit during the study period (*n *= 2,497), 865 (34.64%) were classified as having proteinuria. In the patients whose total burn surface areas (TBSA) were > 30% (*n *= 396), 271 patients (68.43%) had proteinuria and 152 of these patients (56.09%) met AKI criteria. No patients without proteinuria developed AKI. Intensive care unit (ICU) mortality rates were 0.8%, 16.67% and 30.77% (*P *< 0.001) in the groups with no, mild and heavy proteinuria, respectively. Logistic regression analysis identified proteinuria (OR 4.48; 95% CI, 2.824 to 7.108; *P *< 0.001) and sequential organ failure assessment (OR 1.383; 95% CI, 1.267 to 1.509; *P *< 0.001) as risk factors for AKI.

**Conclusions:**

We observed a high prevalence of proteinuria in patients with severe burns (> 30% TBSA). Severely burned patients with proteinuria had a high risk of developing AKI and a poor prognosis for survival. This suggests that proteinuria should be used for identifying burn patients at risk of developing AKI.

## Introduction

Acute kidney injury (AKI) is increasingly common and is associated with adverse short- and long-term outcomes in various clinical settings [1], including patients with severe burns [2]. In the acute phase, as kidney function declines, AKI is associated with excess mortality [3,4], maximum Sequential Organ Failure Assessment (SOFA) and extended intensive care unit (ICU) stays [2,5,6]. Although kidney function recovers for most burn patients survivors [7-10], some survivors have persistent loss of kidney function and require long-term dialysis [11]. The key strategy for prevention of AKI is to identify those at highest risk so that prophylactic measures can be administered.

As defined by the RIFLE (Risk, Injury, Failure, Loss, End Stage) classification system, the diagnosis of AKI is currently based on changes in the serum creatinine (sCr) or urine output [12,13]. However, these parameters often lag behind acute changes in renal function and, therefore, underestimate the degree of renal dysfunction in acute care settings [14]. Recent improvements in understanding AKI have resulted in the implementation of proteinuria [13,15-17] and some novel biomarkers [18-20] as means of more accurately assessing for AKI.

Proteinuria is an indicator of both glomerular and renal endothelial injury in chronic disease [21,22], acute illness or is due to surgery [23-26]. Glomerular proteinuria is a feature of chronic kidney disease (CKD) and intrinsic renal disease, whereas tubular proteinuria occurs more frequently in AKI. Dipstick urinalysis is a cheap and convenient means of diagnosing proteinuria, and it is a routine test for in-patients in our burn center (the Burn Department of Southwest Hospital). Proteinuria is commonly observed in patients with severe burns [27,28], but the usefulness of proteinuria for predicting the risk of AKI and prognosis has not been documented for burn patients. In this study, we examined associations between proteinuria and adverse clinical outcomes, including mortality and AKI incidence in burn patients. We hypothesized that burn patients with proteinuria would be at higher risk of AKI and adverse outcomes than patients without proteinuria.

### Materials and methods

This retrospective study was conducted over a five-year period (October 2006 to September 2010) in a 200-bed specialized burn department. All patients with burn injuries admitted to the Burn Department of Southwest Hospital were included in the study. This study was approved by the Human Subjects Review Board of The Third Military Medical University, and did not require informed consent because it was a study of routinely collected clinical data. All relevant information was provided following the STROBE (STrengthening the Reporting of OBservational studies in Epidemiology) Guideline.

Exclusion criteria for the study were: age < 18 years or > 75 years; non-burn diagnosis (reconstructive surgery); non-survivable burns (decision for comfort care on admission); pregnancy; admission for less than 72 h; previous dialysis; previous abnormal renal function with sCr > 133 μmol/L; or diagnosed with diabetes or hypertension.

Patient resuscitation in the first 48 h was based on the Third Military Medical University formula [29] (see Additional file 1). Thereafter, maintenance crystalloid infusion rates were calculated using standard formulas for insensible wound fluid losses. Urine volumes > 0.5 ml/Kg· h^-1 ^in the first 48 h post injury were considered to be indicative of effective resuscitation. Enteral or intravenous nutrition was provided after the first 48 h post-injury for patients with total burn surface areas (TBSA) > 50%, and prophylactic antibiotics were used for patients with TBSA > 30%. Demographic and clinical data were recorded for each patient, including proteinuria; sCr; TBSA and depth; comorbidities; escharotomies; inhalation, chemical, or electrical injuries; mechanical ventilation; continuous renal replacement therapy (CRRT); abdominal compartment syndrome (ACS); nephrotoxic drugs (vancomycin, amphotericin B, polymyxin E, aminoglycosides); vasopressors (dopamine, epinephrine, norepinephrine); administration of colloidal solutions for resuscitation; urine volume in the first 48 h post injury; ICU length of stay; and mortality. Intra-abdominal pressure (IAP) was measured using once daily urinary bladder pressure measurements, and ACS was defined as a sustained IAP > 20 mm Hg that was associated with development of organ dysfunction/failure [30]. Proteinuria was determined with urine dipsticks using an automated urine analyzer (H800, Dirui, Changchun, China), and defined as normal (negative), mild (± or 1+, approximately > 5 to 20 mg/dL), or heavy (≥ 2+, approximately ≥ 100 mg/dL). The maximal level of proteinuria prior to the first diagnosis of AKI was recorded in AKI patients and the maximum level at any time in non-AKI patients. Urine protein results were recorded by the laboratory examiner, and the urine analyzer was calibrated by the factory engineer every two weeks to ensure accuracy and precision.

Baseline sCr values were measured within three months prior to admission. For the patients without a sCr test within three months, the first sCr value after admission was adopted as the baseline sCr values if the first sCr value was normal (< 106 μM for male, and < 88 μM for female). If the first sCr value was abnormal, patients were assigned to a baseline eGFR of 75 ml/min/1.73 m^2^, and the Modification of Diet in Renal Disease (MDRD) equation was applied [12,31]. The RIFLE class was determined according to the worst degree of either sCr or urine output criteria [12]. Systemic inflammatory response syndrome (SIRS) and sepsis were diagnosed according to criteria defined by the American College of Chest Physicians and the Society of Critical Care Medicine [32]. For the diagnosis of multiple organ dysfunction or failure, Sequential Organ Failure Assessment (SOFA) Scores were computed daily and the maximum was used for this study [33].

### Statistical analysis

Continuous variables were expressed as medians and quartile ranges. Dichotomous and categorical variables were expressed as proportions and compared using non-parametric and chi-square (χ^2^) tests. Comparisons of location parameters for continuous data were analyzed with Mann-Whitney U and Kruskal-Wallis H tests. Risk factors were assessed using a binary logistic regression with proteinuria, AKI and ICU mortality as the dependent outcome variables. In all comparisons, a *P-*value of < 0.05 was considered statistically significant. Statistical analyses were performed with the software program IBM SPSS STATISTIC, version 19 (SPSS Inc, Chicago, IL, USA).

## Results

**Figure 1:**
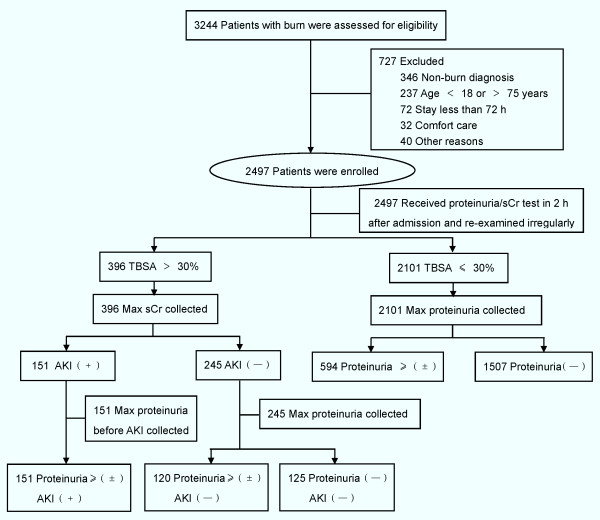
**Study flow**. The details of recruitment and inclusion, and the procedures of data collection.

A total of 3,224 patients were admitted to the Burn Department during the study period, and 2,497 patients met the study's inclusion criteria (Figure 1). Patients were excluded on the basis of non-burn diagnoses (*n *= 346); age < 18 years or > 75 years (*n *= 237); abnormal renal function preoperatively (defined as sCr > 133 μmol/L) or previous dialysis (*n *= 12); patients who stayed less than 72 hours after admission (*n *= 72); patients who elected comfort care upon admission (*n *= 32); and pregnancy (*n *= 28).

All patients had their first measurement of proteinuria and sCr around 2 h after admission. Maximum proteinuria values of the remaining 2,497 patients and maximum sCr values of the 396 patients, whose TBSA > 30%, were recorded, in which maximum proteinuria was defined as the maximum value before AKI incidence for patients with AKI. Patient proteinuria and TBSA values were summarized (Table 1). In the remaining 2,497 patients, 865 (34.64%) developed proteinuria, and the patients with larger TBSA were more likely to have proteinuria (*P *< 0.001).

**Table 1:** Comparison of TBSA with proteinuria (*n *= 2,497)

TBSA (%)	No proteinuria (*n *= 1,632)	Proteinuria (*n *= 865)	Proteinuria (%)
≤ 10	973	324	24.98
11 to 30	533	270	33.62
31 to 50	99	110	52.63
51 to 70	16	67	80.72
> 70	11	94	89.52

TBSA, total burn surface area

**Figure 2:**
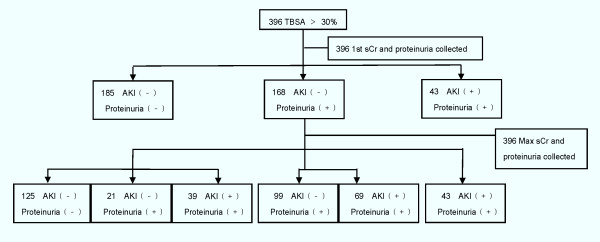
**Flow chart of patients' progress**. The details of acute kidney injury and proteinuria progression.

The difference between the first and maximum proteinuria has been summarized in Figure 2. Of the patients with TBSA > 30% (*n *= 396), 271 patients had proteinuria, in which 211 had proteinuria at their first measurement. A total of 151 patients developed AKI, 43 of which had AKI on admission. It should be specially noted that none of the patients without proteinuria had AKI, which means that the continued absence of proteinuria excludes the development of AKI.

Patient categories were compared based on proteinuria (Table 2). Of the patients with TBSA > 30% (*n *= 396), 180 (45.45%) had mild and 91 (22.98%) had heavy proteinuria. Of the patients with proteinuria (*n *= 271), 151 (55.72%) had AKI, with RIFLE classifications of: Risk (*n *= 83; 54.97%); Injury (*n *= 52; 34.44%); and Failure (*n *= 16; 10.60%). Non-parametric tests (univariate analysis) showed significant differences among the groups for: age, TBSA, ACS, escharotomy, comorbidities, nephrotoxic drugs, inhalation injury, urine output at the shock stage, vasopressor in first 48 h, sepsis during the ICU stay, positive blood culture, maximum SOFA and RIFLE (*P *< 0.05). Patients with proteinuria were prone to having CRRT (*P *= 0.013), longer mechanical ventilation duration (*P *< 0.001), longer ICU stays (*P *< 0.001), and higher mortality rates (0.8%, 16.67% and 30.77% for none, mild and heavy proteinuria) (*P *< 0.001).

**Table 2:** Characteristics and outcomes of patients according to degree of proteinuria (*n *= 396)

	None (*n *= 125)	Mild (*n *= 180)	Heavy (*n *= 91)	*P-*value
Age (years)	38 (16.00)	42 (16.75)	41 (15.00)	0.050
Gender (% of males)	72.80	83.89	86.81	0.150
TBSA (%)	38 (15.50)	54.4 (35.00)	70 (41.00)	< 0.001
dTBSA (%)	35 (13.50)	48 (33.00)	60 (39.00)	< 0.001
Escharotomy (%)	3 (15.50)	9 (27.75)	24 (38.00)	< 0.001
				
Electrical injury (%)	7.20	7.22	10.99	0.511
Chemical injury (%)	24.00	17.88	10.99	0.049
Inhalation injury (%)	25.60	46.67	48.35	< 0.001
Comorbidities (%)	1.60	9.44	7.69	0.042
ACS (%)	3.20	17.78	25.78	< 0.001
Vasopressor in first 48 h (%)	0.80	6.67	7.69	0.030
Artificial colloid (%)	38.46	53.72	53.85	0.341
Albumin (%)	57.69	34.71	32.05	0.540
Urine output at shock stage (%, ≥ 0.5 ml/Kg·h^-1^)	97.60	75.00	64.84	< 0.001
Nephrotoxic drugs (%)	23.08	51.24	55.13	0.015
Length of mechanical ventilation (days)	0 (0.00)	0 (2.00)	1 (6.00)	> 0.001
Sepsis during ICU stay (%)	15.20	46.11	67.03	< 0.001
Positive blood culture (%)	54.51	58.37	87.12	< 0.001
Gram-positive bacteria (%)	9.68	51.61	38.71	< 0.001
Gram-negative bacteria (%)	23.53	50.00	26.47	< 0.001
Positive culture, others (%)	0.00	51.56	48.44	< 0.001
Maximum RIFLE				< 0.001
No AKI (%, *n *= 245)	51.02	36.73	12.24	< 0.001
Risk (%, *n *= 83)	0.00	61.45	38.55	< 0.001
Injury (%, *n *= 52)	0.00	61.54	38.46	< 0.001
Failure (%, *n *= 16)	0.00	43.75	56.25	< 0.001
				
				
Maximum SOFA	0 (0.00)	4 (7.00)	6 (8.00)	< 0.001
CRRT during ICU stay (%)	0.00	5.00	7.69	< 0.001
Length of CRRT treatment (days)	0 (0.00)	0 (0.00)	0 (2.00)	0.013
ICU length of stay (days)	0 (0.00)	2 (30.00)	4 (49.00)	< 0.001
ICU mortality (%)	0.8	16.67	30.77	< 0.001

ACS, abdominal compartment syndrome; CRRT, continuous renal replacement therapy; dTBSA, total surface area of deep second degree or third degree burn; ICU, intensive care unit; RIFLE, Risk, Injury, Failure, Loss, End Stage classification; SOFA, sequential organ failure assessment; TBSA, total burn surface area. Continuous variables, median (quartile range); categorical variables, (%).

Variables coding: Maximum RIFLE 1: No-AKI, 2: Risk, 3: Injury, 4: Failure + loss + end stage

The characteristics and outcomes of patients with proteinuria according to AKI were summarized (Table 3). Among the 396 patients with TBSA > 30%, 357 had baseline sCr values, whereas the remaining 41 used eGFR derived values. When diagnosing the AKI, 102 achieved RIFLE maximum by sCr criteria, 19 by urine criteria and 31 by both criteria. The patients with AKI had higher TBSA, ACS, escharotomy, proteinuria, sepsis during ICU stay and maximum SOFA values compared to the patients without AKI (*P *< 0.05). Patients with AKI were prone to have CRRT (*P *= 0.002), longer mechanical ventilation durations (*P *< 0.001), and extended ICU stays (*P *< 0.001). Mortality rates of patients with and without AKI were 12.50% and 28.48% (*P *= 0.001), respectively.

**Table 3:** Characteristics and outcomes of patients with proteinuria according to AKI (*n *= 271)

	Proteinuria without AKI (*n *= 120)	Proteinuria accompanied with AKI (*n *= 151)	*P-*value
Age (years)	41 (17.75)	41 (16.00)	0.685
Gender (% of male)	72.80	79.17	0.200
TBSA (%)	47.5 (34.75)	70 (35.00)	< 0.001
dTBSA (%)	42 (25.75)	58 (37.00)	< 0.001
Escharotomy (%)	7 (28.50)	18 (38.00)	< 0.001
			
			
			
Electrical injury (%)	8.33	8.61	0.936
Chemical injury (%)	19.17	12.58	0.138
Inhalation injury (%)	43.33	50.33	0.226
Comorbidities (%)	10.00	8.95	0.630
ACS (%)	14.17	25.17	0.033
Vasopressor in first 48 h (%)	5.00	8.61	0.249
Artificial colloid (%)	56.14	52.82	0.672
Albumin (%)	42.11	30.28	0.111
Proteinuria at heavy stage (%)	22.71	42.14	0.001
Urine output in shock stage (%, ≥ 0.5 ml/Kg·h^-1^)	77.19	54.93	< 0.001
Nephrotoxic drugs (%)	45.61	55.63	0.202
Length of mechanical ventilation (days)	0 (3.00)	1 (6.00)	< 0.001
Sepsis during ICU stay (%)	41.67	62.25	0.001
Positive blood culture (%)	71.28	74.35	0.102
Maximum SOFA	0 (5.00)	7 (9.00)	< 0.001
CRRT during ICU stay (%)	8.30	9.93	0.002
Length of CRRT treatment (days)	0 (0.00)	0 (2.00)	0.002
ICU length of stay (days)	0 (24.75)	11 (45.00)	< 0.001
ICU mortality (%)	12.50	28.48	0.001

ACS, abdominal compartment syndrome; AKI, acute kidney injury; CRRT, continuous renal replacement therapy; ICU, intense care unit; SOFA, sequential organ failure assessment; TBSA, total burn surface area; tTBSA, total surface area of third degree burn. Continuous variables, median (quartile range); categorical variables, (%)

Logistic regression analysis identified TBSA (OR 1.038; 95% CI, 1.022 to 1.054; *P *< 0.001), sepsis (OR 1.052; 95% CI, 1.539 to 5.331; *P *= 0.001), and age (OR 1.026; 95% CI, 1.007 to 1.046; *P *= 0.006) as independent risk factors for proteinuria (Table 4); SOFA (OR 1.383; 95% CI, 1.267 to 1.509; *P <*0.001) and proteinuria (OR 4.480; 95% CI, 2.824 to 7.108; *P *< 0.001) as independent risk factors for AKI (Table 5); age (OR 1.085; 95% CI, 1.048 to 1.123; *P *< 0.001), TBSA (OR 1.053; 95% CI, 1.032 to 1.075; *P *< 0.001), proteinuria (OR 1.932; 95% CI, 1.000 to 3.734; *P *= 0.050), associated injury (OR 2.446; 95% CI, 3.986 to 33.440; *P *< 0.001) and RIFLE (OR 2.289; 95% CI, 1.577 to 3.322; *P *< 0.001) as independent risk factors for ICU mortality (Table 6).

**Table 4:** Logistic regression model: risk factors for proteinuria (*n *= 396)

Covariates associated with proteinuria	Coefficient	Odds ratio	95% Confidence interval	*P-*value
TBSA (%)	0.037	1.038	1.022 to 1.054	< 0.001
Sepsis during ICU stay	1.052	2.864	1.539 to 5.331	0.001
				
				
				
				
Age	0.026	1.026	1.007 to 1.046	0.006

ICU, intense care unit; TBSA, total burn surface area

Covariates selected: Age, TBSA, ACS, inhalation injury, vasopressor in the first 48 h, comorbidities, albumin, sepsis during ICU stay

Variables coding: Proteinuria 1: (-), 2: ≥ (±)

**Table 5:** Logistic regression model: risk factors for AKI (*n *= 396)

Covariates associated with AKI	Coefficient	Odds ratio	95% Confidence interval	*P-*value
Maximum SOFA	0.324	1.383	1.267 to 1.509	< 0.001
Proteinuria	1.500	4.480	2.824 to 7.108	< 0.001

SOFA, sequential organ failure assessment

Covariates selected: Age, TBSA, ACS, inhalation injury, vasopressor in the first 48 h, comorbidities, sepsis during ICU stay, maximum SOFA, proteinuria

Variables coding: AKI 1: No occurrence of RIFLE criteria, 2: Occurrence of RIFLE criteria; Proteinuria 1: (-), 2: (±) or (+), 3: ≥ (++)

**Table 6:** Logistic regression model: risk factors for ICU mortality (*n *= 396)

Covariates associated with ICU mortality	Coefficient	Odds ratio	95% Confidence interval	*P-*value
Proteinuria	0.659	1.932	1.000 to 3.734	0.050
Maximum RIFLE	0.828	2.289	1.577 to 3.322	< 0.001
Comorbidities	2.446	11.545	3.986 to 33.440	< 0.001
Age	0.065	1.068	1.029 to 1.108	< 0.001
				
TBSA	0.462	1.587	1.421 to 1.772	< 0.001

RIFLE, Risk, Injury, Failure, Loss, End Stage classification;

SOFA, sequential organ failure assessment; TBSA, total burn surface area. Covariates selection: Age, TBSA, ACS, inhalation injury, vasopressor in the first 48 h, comorbidities, sepsis during ICU stay, proteinuria, maximum RIFLE

Variables coding: Proteinuria 1: (-), 2: (±) or (+), 3: ≥ (++); Maximum RIFLE: 1: No-AKI, 2: Risk, 3: Injury, 4: Failure + loss + end stage

There was no interaction between proteinuria and RIFLE.

## Discussion

In this hospital-based study, we found that proteinuria was common (68.26%) in severe burn patients (> 30% TBSA), and proteinuria should be used to identify severe burn patients at risk of AKI, as well as for prognosis and identification of burn patients needing intensive care.

Acute kidney injury is characterized as a rapid loss of kidney function which clinically manifests as an abrupt and sustained rise in urea and creatinine. Life threatening consequences of AKI include volume overload, metabolic acidosis, hyperkalaemia and effects on other organ systems [34]. AKI is associated with increased morbidity, mortality and hospitalization costs [19]. So it is significantly meaningful to identify the patients at risk of AKI and take appropriate treatments. However, decreased creatinine production and hemodilution will significantly blunt increases in creatinine that could be indices of AKI in burn patients. Furthermore, some patients with proteinuria may die before they are diagnosed with AKI.

It is established that the risks of death and AKI progression, or end-stage renal disease associated with AKI, vary with levels of proteinuria [10,35]. Proteinuria in the absence of AKI is common in severe burn patients and may indicate sub-clinical AKI. However, current guidelines for the classification and staging of AKI do not explicitly consider concomitant proteinuria [13] and, therefore, may not identify individuals at risk for developing AKI when sCr is normal but proteinuria exists.

Dipstick urinalysis was used for measurement of proteinuria in our study. Dipstick urinalysis has less favorable diagnostic properties for the assessment of proteinuria than quantitative measurements of proteinuria, such as 24-hour urine collection tests. However, it is the most common test and is much cheaper, readily available, feasible and practical, compared with automated measures. The validity of proteinuria readings was established with a significant correlation between dipstick and 24-hour urine collection testing using bivariate correlation analysis (*n *= 40, *P <*0.001; data not shown). Although our findings do not address whether dipstick urinalysis or urine sample measurement of albumin is preferable for risk assessment in clinical practice, they do suggest that for burn patients, testing of proteinuria using simple dipstick methodology is prognostic for AKI outcomes.

The majority of proteinuria in AKI is due to tubular injury that prevents protein absorption, as well as release of tubular proteins into the ultrafiltrate. However, kidney biopsies of eight patients with persistent AKI/proteinuria documented co-existent glomerular disease (data not shown). This suggests that the role of glomerular injury in AKI, particularly where there is sepsis and inflammation, is incompletely understood and may be of greater significance than currently recognized.

Intrinsic AKI is often multifactorial. Renal ischemia is probably less important in the acute phase of burn injury than originally presumed [36]. Instead, inflammation and sepsis probably play important roles [37-39]. An important difference between burn and other types of ICU patients may be the intensity and duration of the inflammatory response, which may persist longer than in other trauma patients [40]. Sepsis in severe burn patients, often accompanied by multi-organ failure (represented by SOFA), was identified as a risk factor for proteinuria by our regression analysis (Table 4), as well as for AKI (Table 5). Sepsis-related inflammatory and thrombogenic factors are considered risk factors for direct renal parenchymal damage [41,42].

This observational study has limitations. First, the burn patients were primarily male, and misclassifications of baseline kidney function could have occurred because of deficiencies in previous examinations. Second, misclassifications of proteinuria might occur because of known variability of urine dipstick measurements, even though we established a significant correlation between dipstick measurements and the 24 h urine test. Finally, we could not exclude the possibility of residual confounding due to burn severity, blood pressure, large amounts of fluid resuscitation and the administration of drugs. Furthermore, the unusual composition of urinary proteins (decreased filtered load of albumin, increased load of acute phase reactants or alterations in protein processing in renal tubules) compromises the use of urinary protein measurements for detecting kidney injury in burn patients [27]. Nonetheless, our results were consistent for several clinically relevant outcomes, including AKI, CRRT and mechanical ventilation duration, ICU length of stay and mortality. These findings are important because current guidelines for the classification and staging of AKI are based on sCr and urine volume, without explicit consideration of the severity of concomitant or independently existing proteinuria, especially in severe burn patients.

## Conclusions

In conclusion, proteinuria was common (68.26%) in severe burn patients (> 30% TBSA), and proteinuria was clearly associated with increased risk for developing AKI, long-term ICU stays and mortality. Age, TBSA and sepsis were considered as independent risk factors for proteinuria in the post-burn period, and maximum SOFA and proteinuria as risk factors for AKI. Age, TBSA, proteinuria, comorbidities and RIFLE were regarded as risk factors for ICU mortality. These findings demonstrate that proteinuria should be used to identify severe burn patients at risk of developing AKI and unfavorable clinical outcomes.

## Key messages

• Almost two-thirds of severe burn patients (> 30% TBSA) develop new onset proteinuria.

• Proteinuria is associated with increased risk for AKI, ICU length of stay and mortality.

• Age, TBSA and sepsis are considered as independent risk factors for proteinuria in the post-burn period.

• Maximum SOFA and proteinuria are regarded as risk factors for AKI.

## Abbreviations

ACS: abdominal compartment syndrome; AKI: acute kidney injury; CKD: chronic kidney disease; CRRT: continuous renal replacement therapy; GFR: glomerular filtration rate; IAP: intra-abdominal pressure; ICU: intense care unit; MDRD: Modification of Diet in Renal Disease; MODS: multiple organ dysfunction syndrome; RIFLE classification: Risk, Injury, Failure, Loss, End Stage classification; sCr: serum creatinine; SIRS: systemic inflammatory response syndrome; SOFA: sequential organ failure assessment; TBSA: total burn surface area.

## Competing interests

The authors declare that they have no competing interests.

## Authors' contributions

HJY participated in conception, design, data analysis and manuscript drafting. MXC and LQZ participated in conception, design, data acquisition and interpretation, and critical revision of the manuscript. HJ was involved in data analysis and drafting the manuscript. HYS was involved in revising the manuscript for important intellectual content, and supervision. PYZ and WJ participated in the study design and critically revised the manuscript for important content. XF and FYD participated in collecting data and coordination. WYZ participated in statistical analysis and revision of the manuscript for important intellectual content. All authors read and approved the final manuscript.

## Supplementary Material

Additional file 1 **The Third Military Medical University formula**. A description of the Third Military Medical University formula for patient resuscitation. Click here for file
