# Supplementary material for: Relation between proteinuria and acute kidney injury in patients with severe burns
Source: Crit Care. 2012 Sep 29;16(5):R172. doi: 10.1186/cc11649 (PMC3682271; doi:10.1186/cc11649)
Supplement: Additional file 1 — The Third Military Medical University formula. A description of the Third Military Medical University formula for patient resuscitation. [file cc11649-S1.DOC]

**Supplementary Material**

The TMMU formula is as follows:

Fluid for first 24 hours (ml) = 1.5×% BSA × Patient's weight in kg, of a crystalloid / colloidal solution at a 2:1 ratio, with the addition of 2000 ml water. The fluid is sequentially delivered (in the following order: crystalloid, colloidal solution, water) with the first half delivered in the first 8 hours, and the remaining half delivered in the following 16 hours.

Fluid for second 24 hours (ml) = 1.0 × %BSA × Patient's weight in kg, of a crystalloid / colloidal solution at a 1:1 ratio, with the addition of 2000 ml water. This amount is delivered at a constant rate for 24 hours.

The percentage of burn body surface area (%BSA) is estimated by the "Rule of 9's," and only the II° and III° burn body surface area is enrolled in the formula.

Crystalloid solution： Lactated Ringer's solution; 0.9% NaCl + 1.25% NaHCO3 (7 : 3).

Colloid solution： [albumin](app:ds:albumin)、frozen fresh plasma、Hydroxyethyl starch 130/0.4 and sodium chloride injection、Succinylated gelatine injection.
